# Self-Reported Levels of Personality Functioning from the Operationalized Psychodynamic Diagnosis (OPD) System and Emotional Intelligence Likely Assess the Same Latent Construct

**DOI:** 10.1080/00223891.2020.1775089

**Published:** 2020-07-06

**Authors:** Emanuel Jauk, Johannes C. Ehrenthal

**Affiliations:** 1 Clinical Psychology and Behavioral Neuroscience, Technische Universität Dresden, Dresden, Germany; 2 Department of Psychology, University of Graz, Graz, Austria; 3 Institute of Medical Psychology, Heidelberg University, Heidelberg, Germany

## Abstract

We tested the hypothesis that structural integration, as assessed in the Operationalized Psychodynamic Diagnosis (OPD) system, and emotional intelligence (EI), as studied in personality psychology, might be closely related constructs at a general level, as both might assess general personality functioning. In three studies (*n*_1_ = 166, *n*_2_ = 204, *n*_3_ = 349), we used a self-report measure of OPD structural integration and measures of Trait and Ability EI. Structural integration and Trait EI display very high correlation at general factor level (*r* = .77 - .82) and almost perfect latent correlation (*r* = .85 - .90). This correlation cannot be explained away by the general positivity of self-views or socially desirable responding. There is also substantial latent correlation between structural integration and Ability EI (*r* = .20 - .65). Results replicate over different samples from different countries and extend to the DSM-5 self-report personality functioning scale.

Contemporary models for the assessment of personality disorders conceive general personality functioning, in terms of basic self- and other-related emotion processing and regulation, as a fundamental ability underlying specific traits. The DSM-5 Alternative Model for the assessment of personality disorders (AMPD) suggests a hybrid approach by employing a measure of personality functioning in the areas of self- and interpersonal functioning, before specifying clinically relevant trait facets (Bender et al., 2011), and a similar proposal has been made for the ICD-11 (Tyrer et al., 2019). While these models have received a considerable amount of attention, most of the existing studies are limited to comparing the new measures to established constructs from psychiatry and clinical psychology. Multitrait-multimethod-designs might help to reduce overestimation of validity due to the influence of shared methods (Zimmermann et al., 2019). Here, we investigate the hypothesis that personality functioning, as conceptualized from a clinical perspective in the Operationalized Psychodynamic Diagnosis (OPD) system, might assess a similar or even isomorphic construct as does emotional intelligence, rooted in nonclinical social/personality psychology.

## OPD levels of structural integration axis (LSIA)

The concept of general personality functioning has a long history in psychodynamic traditions, where it is also referred to as structural integration. The OPD system provides a comprehensive assessment of structural integration, which has been put to test for about 25 years. The system comprises five axes allowing to assess diagnosis according to DSM or ICD (Axis V), experience of illness and treatment prerequisites (Axis I), repetitive maladaptive interpersonal relations (Axis II), motivational conflicts (Axis III), and personality functioning, i.e. “structure” (Axis IV; Ehrenthal & Benecke, 2019; OPD Task Force, 2008). The Levels of Structural Integration Axis (Axis IV; LSIA) integrates theories from ego-psychology, self-psychology, object relations theory, and developmental psychology (Rudolf et al., 2002). It describes psychic structure as core capacities comprising perception/cognition, regulation, communication, and attachment; each directed toward the self and others. Each dimension consists of three facets, leading to a total of 24 facets (see Table 1). The LSIA allows rating each facet as well as an overall score on high, medium, low, and disintegration by trained clinicians or researchers. Research on LSIA expert-ratings comprises more than 17 independent samples including over 2000 participants. Generally, interrater reliability is adequate to high, and a number of studies show construct validity related to psychopathology and personality disorders (Doering et al., 2014; Zimmermann et al., 2012). LSIA severity-ratings are significantly related to DSM-5 AMDP Levels of Personality Functioning Scale severity ratings (Zimmermann et al., 2012, 2014).

In 2012, Ehrenthal and colleagues (Ehrenthal et al., 2012) published a self-report questionnaire to complement the existing LSIA expert-ratings with patients’ perspective. The OPD Structure Questionnaire (OPD-SQ) consists of 95 Items capturing 20 of the original 24 facets, and integrating another two into a single subscale. In the original evaluation with 1112 participants, internal consistency was very high for the total score, and acceptable to high for the subscales. OPD-SQ values displayed strong positive associations with general psychopathology, attachment insecurity, neuroticism, and negative correlations with openness, agreeableness, extraversion, and in part with conscientiousness. There were large differences between individuals in inpatient vs. outpatient vs. no psychotherapy sample, and between individuals with vs. without a diagnosis of a comorbid personality disorder. Notably, nearly all effects still held when statistically controlling for general psychopathology.

The OPD-SQ correlates with OPD LSIA expert-ratings at *r* = .62 (Dinger et al., 2014). It is also highly related to other established questionnaires such as the Borderline Personality Inventory (BPI), the Inventory of Personality Organization (IPO; König et al., 2016), and the General Assessment of Personality Disorder (GAPD; Zimmermann et al., 2015). Zettl and colleagues (2019) found a short form of the OPD-SQ to correlate at *r* = .78 with personality functioning as measured by the Semi-Structured Interview for Personality Functioning DSM-5 (STiP-5.1) as well as reflective functioning as measured by the Reflective Functioning Scale (*r* = −.40), even when controlling for symptom severity (*r* = −.26; Zettl et al., 2020). Further validity evidence shows that the OPD-SQ is associated with stress and burnout in students (Bugaj et al., 2016), quality of life in depressed patients (Crempien et al., 2017), affective experiencing in depressed patients with vs. without borderline personality disorder (BPD; Dinger et al., 2019; Köhling et al., 2016), differs between patients with chronic pain and controls (Albrecht et al., 2015), and is related to changes in plasma glucose in diabetes patients (Ehrenthal et al., 2019).

Taken together, the OPD LSIA is conceptually and empirically related to DSM-5 AMPD LPFS and the ICD-11 proposal for the assessment of personality disorders, and can be conceived a measure of general personality functioning. Ample research supports reliability and validity. However, as with many clinical instruments, there is a lack of cross-validation with concepts from other areas of psychology. To this end, an area of special interest is the field of emotional intelligence, which bears striking conceptual similarities to clinical models of personality functioning.

## Emotional intelligence

Emotional intelligence (EI), originating in social/personality and organizational psychology, intends to describe variance in the ability to attend to, express, process, and utilize affectladen information of intra- and interpersonal origin (Mayer & Salovey, 1997). The concept was introduced in academic psychology by Salovey and Mayer (1990), who advocated the status of EI as a mental ability, paralleling cognitive intelligence (Mayer et al., 1999), and was popularized by Goleman’s (1995) book *Emotional Intelligence*: *Why It Can Matter More Than IQ*. Research on EI sorts into two traditions, which either conceive EI as an ability, sensu Salovey and Mayer, or as a trait, thus referred to as *Ability EI* and *Trait EI*.^1^

## Emotional intelligence as an ability

Ability EI was defined by Salovey and Mayer as “the ability to monitor one’s own and others’ feelings and emotions, to discriminate among them and to use this information to guide one’s thinking and actions” (1990, p. 189). They later expanded the definition to a more comprehensive framework spanning also generative emotional processes and personal growth:

the ability to perceive accurately, appraise, and express emotion; the ability to access and/or generate feelings when they facilitate thought; the ability to understand emotion and emotional knowledge; and the ability to regulate emotions to promote emotional and intellectual growth (Mayer & Salovey, 1997, p. 10).

Given the conception of EI as an ability, researchers strived for an objective, performance-based assessment, which resulted in the construction of the Mayer-Salovey-Caruso Emotional Intelligence Test (MSCEIT; see Mayer et al., 2003). The MSCEIT assesses four hierarchically layered branches of EI, from perceiving emotions and using emotions to facilitate thought to understanding and managing of emotions (Mayer, Salovey, et al., 2008; Mayer & Salovey, 1997). The hierarchical structure implies that more basic emotional processes are necessary for more complex ones (Mayer & Salovey, 1997). The MSCEIT predicts an array of relevant real-life criteria such as peer-rated quality of social interactions (Lopes et al., 2004), social competence in interactions (Brackett et al., 2006), or academic and vocational achievement (for a review, see Mayer, Roberts, et al., 2008) and is also related to mental health and depression in clinical and nonclinical samples (for a review, see Fernández-Berrocal & Extremera, 2016).

More recently, alternative measures have been developed for the assessment of Ability EI, namely the Situational Test of Emotional Understanding (STEU) and the Situational Test of Emotion Management (STEM; Austin, 2010; MacCann & Roberts, 2008). Both show convergent validity (Libbrecht & Lievens, 2012) and criterion validity with respect to well-being (Burrus et al., 2012), anxiety, stress, and aspects of alexithymia (negatively; MacCann & Roberts, 2008), academic achievement (MacCann et al., 2011), or communication skills and interpersonal sensitivity (Libbrecht et al., 2014). In studies 2 and 3, we used the STEM as an overall indicator of Ability EI, given that emotion management also draws upon more basic emotional abilities (Mayer & Salovey, 1997).

## Emotional intelligence as a trait

Trait EI is typically assessed using self-report questionnaires, such as the Emotional Quotient Inventory (Bar-On, 1997) or the Trait Emotional Intelligence Questionnaire (TEIQUE; Petrides & Furnham, 2004). They measure self-perceptions of intra- and interpersonal emotion-related abilities or competencies, which is why Trait EI is also referred to as “trait emotional self-efficacy” (e.g., Petrides et al., 2016, p. 335). Trait EI measures have high reliability and validity with respect to peer-rated prosocial behavior (Mavroveli et al., 2009), relationship satisfaction (for meta-analysis, see Malouff et al., 2014), job performance (for meta-analysis, see O’Boyle et al., 2011), or mental, psychosomatic, and physical health (for meta-analysis, see Martins et al., 2010).

Interestingly, the meta-analytically derived relations with health indicators are stronger for Trait than Ability EI measures (Martins et al., 2010). It is argued that trait measures can be advantageous compared to ability measures (e.g., Petrides, 2011), as they assess *typical* rather than *maximal* performance (Freudenthaler & Neubauer, 2007; Freudenthaler & Neubauer, 2005). While Ability EI tests capture how an individual can *potentially* behave, Trait EI measures capture how they *usually* behave. Thus, the correlation between measures of Trait and Ability EI is typically low to moderate (*r* ~ .20 - .40; e.g. Austin, 2010; Brannick et al., 2009).

In studies 1 and 3, we used the TEIQUE to measure Trait EI. The sampling domains of the TEIQUE comprise the factors *well-being*, *self-control*, *emotionality*, and *sociability* and their respective facets (see Table 1; Petrides et al., 2016). Trait EI is conceived a distinct yet compound factor within the personality space (Petrides et al., 2007). It is highly correlated (*r* = .85) with the *general factor of personality* (GFP; shared variance among Five-Factor Model dimensions; Musek, 2007; van der Linden et al., 2017), which, in turn, also relates to Ability EI (*r* = .28; van der Linden et al., 2017). Taken together, Trait EI can be conceived a common fundamental dimension underlying more differentiated personality traits, making it conceptually similar to structural integration or personality functioning (criterion A in the DSM-5 AMPD).

## Structural integration and emotional intelligence: A jangle fallacy?

From the reviewed literature, major overlaps between the concepts of structural integration (or more generally, personality functioning) and EI become apparent. Though emerging from different research traditions, both circumscribe fundamental emotional competencies, such as being attentive to own and others’ emotions, expressing and interpreting them in an adequate manner, and adjusting intra- and interpersonal reactions accordingly, which contribute to intra- and interpersonal social functioning.

As an example, the LSIA facets *affect differentiation* and *self-object differentiation* (self- and object perception) as well as *experiencing* and *communicating affect* (internal and external communication; OPD Task Force, 2008) might relate to the factor *emotion perception (self and other)*, circumscribed as “clear about their own and other people’s feelings”, and *emotion expression* (“capable of communicating their feelings to others”) in the TEIQUE Trait EI model (Petrides et al., 2016, p. 336), as well as *perception*, *appraisal*, *and expression of emotion* in the Ability EI model (Mayer & Salovey, 1997). As another, more high-level example, the LSIA dimension *self-regulation* (including the facet impulse control) might relate to the Trait EI facet *impulse control* (“reflective and less likely to give into their urges”; (Petrides et al., 2016, p. 336), and aspects of the Ability EI *reflective regulation* branch (“ability to reflectively engage or detach from an emotion”; Mayer & Salovey, 1997, p. 11).

Though the concepts are partially expressed in different vocabularies, particularly regarding high-level functions, it can be hypothesized that the underlying dimensions are overlapping to a high degree. In this case, the existence of two independent research traditions could be considered an instantiation of a Jangle Fallacy – the assumption that two constructs differ because they are labeled differently (Kelley, 1927). Importantly, while this does not imply that all aspects of the constructs are interchangeable, the constructs might still have more in common than divides them. If so, this might enable a rich transfer of knowledge between two largely unrelated research traditions.

## Research aim and rationale

We tested the hypothesis that structural integration and EI are closely related constructs at a general level. We conducted three studies in which we tested the relations of selfreport level of structural integration (OPD-SQ) with Trait EI (TEIQUE; study 1), Ability EI (STEM; study 2), and replicated these in a community sample, also relating both constructs to the DSM-5 AMPD self-report inventory (study 3).

In study 1, we hypothesized that there will be a high degree of correspondence between OPD-SQ and TEIQUE general factor scores; possibly in the range of correlations between the GFP and Trait EI (latent *r* ~ .85; van der Linden et al., 2017). We also expected substantial correlations between the single factors of the OPD-SQ and the TEIQUE, though, due to the different structures underlying both models, these should not be as high. To control for the possibility that the correlation between OPD-SQ and TEIQUE general factor scores can be accounted for by the general positivity of self-views (e.g., Leising et al., 2015), we applied statistical correction. Finally, we tested the unique and common contributions (commonality analyses) of OPD-SQ and TEIQUE scores to variance explained in selected criteria of personality, psychological adjustment, and experienced adversity (Dark Triad traits, unusual experiences, perceived social support, life satisfaction, and adverse childhood experiences). We hypothesized that there would be substantial shared effects, and beyond that, OPD-SQ scores would have higher unique contributions to measures of maladaptive adjustment, whereas TEIQUE scores would have higher contributions for adaptive adjustment. In study 2, we hypothesized that there will be a low to moderate association of OPD-SQ structural integration and STEM emotion management as an indicator of Ability EI. We base this prediction on previous works on the association between Trait and Ability EI (*r* ~ .20 - .40; e.g. Austin, 2010; Brannick et al., 2009). In study 3, we aimed to replicate the relationships in a community sample, and extend them to the DSM-5 AMPD personality functioning scale as well as non-clinical and clinical Five-Factor Model scales.

## Study 1

### Method

#### Participants and procedure

A total of *N* = 166 (83 male, 83 female) participants took part in study 1. The sample size was chosen in order to detect small to moderate effects (*r* > .20) in zero-order correlations at a power of 1 – *β* = .8 and to allow for the estimation of structural equation models (*N* ~ 200 or at least 5 per estimated parameter; Kline, 2011). The mean age of the sample was 24.73 years (*SD* = 4.88; range 19 – 40). All were either native German speakers (94.6%) or had sufficient German skills (assessed by the experimenters; see below) to take part in the study. Concerning educational status, 1.8% had less than 12 years of schooling, 79,5% had at least 12 years of schooling or professional education, and 18.1% had a university degree (one person did not disclose educational status). 27% of the sample were students of psychology, the rest had diverse study majors or professions. Participants gave written informed consent; the protocol was approved by the IRB of the University of Graz (Austria).

Participants were approached by students of the University of Graz and assessed using standardized test booklets. An experimenter was present during the whole assessment. As the study was part of a larger project on personality, psychological adjustment, personal relationships, and creativity, participants completed several questionnaire and performance measures of personality, trauma, sexuality, life satisfaction and creativity. The scales that are of relevance to the present analysis are described in detail below. The order of assessment was: OPD-SF, measures of psychological adjustment, TEIQUE, Dark Triad, unusual experiences, self-esteem, adverse experiences. The duration of the assessment was about one hour. The percentage of missing data was low, not exceeding 2.5% (*n* = 4) on any single item. Data can be obtained from the Open Science framework: https://osf.io/dcmeq/.

### Measures

#### OPD structural integration

The OPD Structure Questionnaire (OPD-SQ; Ehrenthal et al., 2012) assesses the self-report level of structural integration using 95 German items. The OPD-SQ yields a general factor score, eight subfactor scores, and 21 facets. The OPD-SQ is structured in a hierarchical manner with eight subfactor scores corresponding to self- and other-related functioning in four domains (see Table 1). The internal consistency of the overall score was *α* = .94, reliabilities for the eight subfactor scores ranged from *α* = .69 to *α* = .86. Higher values correspond to higher degrees of structural impairment in the original OPD-SQ. We adopt this scoring for the presentation of mean values to ensure comparability to previous studies. For correlational analyses, we reversed the scores so that higher scores reflect higher structural integration. This parallels the scoring of the TEIQUE (see below) and thus facilitates the interpretation of correlations.

#### Trait emotional intelligence

We assessed Trait EI using the Trait Emotional Intelligence Questionnaire (TEIQUE; Petrides, 2009), German Version by Freudenthaler et al., 2008). The 153-item questionnaire yields an overall score and four subfactors scores (see Table 1). The reliability of the general factor score was *α* = .95, the subfactors ranged from *α* = .82 to *α* = .93, the facets ranged from *α* = .56 to *α* = .91.

### Personality

#### Self-esteem

We used the German Multidimensional Self-Esteem scale (Schütz et al., 2016) as an indicator of overall self-esteem. The 32-item scale assesses global self-esteem across the eight domains *emotion*, *social contact*, *dealing with critique*, *performance*, *physical attractiveness*, and *sportiness*. The overall internal consistency of the scale was *α* = .95, all domain-scales displayed internal consistencies > *α* = .81.

#### Dark triad traits

We assessed the personality traits *narcissism*, *Machiavellianism*, and *psychopathy* using the 12-item Dark Triad dirty dozen scale (Jonason & Webster, 2010; German version by Küfner et al., 2015). The three scales displayed good to acceptable internal consistencies (narcissism: *α* = .83, Machiavellianism: *α* = .81, psychopathy: *α* = .69), comparable to previous studies (Küfner et al., 2015).

#### Unusual experiences

To assess unusual (psychotic) experiences that might be related to lower levels of structural integration, we used the unusual experiences subscale of the German Oxford-Liverpool Inventory of Feelings and Experiences (O-LIFE; Mason & Claridge, 2006; German version by Grant et al., 2013). The scale displayed acceptable internal consistency (*α* = .74).

### Psychological adjustment

#### Perceived social support

We used a German translation of the Multidimensional Scale of Perceived Social Support (MSPSS; Zimet et al., 1988). The 12-item scale may be analyzed separately for significant other, family, and friend subscales. However, for the present study, we focused on the overall score. The internal consistency of the overall score (*α* = .90) was similar to the original (Zimet et al., 1988).

#### Life satisfaction

As an indicator of general psychological adjustment, we used a brief 5-item measure of life satisfaction (Satisfaction with Life Scale, SWLS; Diener et al., 1985, German version by Schumacher, 2003). The scale displayed good internal consistency (*α* = .84).

### Adverse childhood experiences

To assess traumatic childhood experiences, we used a German translation of the World Health Organization’s Adverse Childhood Experiences International Questionnaire (ACE-IQ; World Health Organization, 2018). It measures the frequency of 13 different adverse experiences, for instance *physical*, *emotional*, or *sexual abuse*, or *chronically mentally ill household member*. The ACE-IQ can be scored according to a binary scheme (did an adverse event ever happen?) or to a frequency scheme (did an adverse event frequently happen?). The binary and frequency scoring correlated *r* = .79. As the binary scoring yielded a less skewed distribution and appears to be more adequate for non-clinical samples, we used this score. One participant did not fill out the ACE-IQ.

### Analysis strategy

To investigate the similarities and differences in the nomological networks of structural integration and EI, we first analyzed correlations between the OPD-SQ and the TEIQUE as well as measures of personality and psychological adjustment at the level of general factors and subfactors. As we observed a very high correlation between the OPD-SQ and the TEIQUE, we next investigated the potentially confounding influence of the global positivity of selfviews. We used self-esteem as an indicator of the global positivity of self-views (cf. Leising et al., 2015). We conducted a partial correlation analysis at manifest level, and set up a structural equation model controlling for selfesteem at latent level.

We used latent variable structural equation models to investigate (a) the latent correlations between structural integration and EI and (b) to test for the effect of the global positivity of self-views. As we were interested in the latent correlation at the highest hierarchical level, we modeled general factors of both constructs using four indicators each (OPD-SQ: perception, regulation, communication, attachment; TEIUQE: well-being, self-control, emotionality, sociability). These reflect the intended theoretical factor structure of the respective constructs and are symmetrical with respect to the number of indicators. We used a two-step modeling procedure, in which single measurement models are evaluated first. This ensures that measurement models are adequately specified before entering them into a larger structural model (Anderson & Gerbing, 1988).

We used self-esteem (modeled by the six subscales of the scale) to control for the positivity of self-views (Leising et al., 2015) and thus test whether structural integration and EI would still be substantially related once evaluative aspects are held constant. Note that this is a very strict test of convergent validity of the two constructs, as both include level and regulation of self-esteem on a conceptual basis.

Lastly, we were interested in the shared and unique portions of variance explained by the two constructs in validity measures. We conducted commonality analyses, which allow disentangling the unique and shared effects of a given predictor set (Nimon & Oswald, 2013). We hypothesized that the two constructs would display largely overlapping effects, and that the structural integration measure would relate more to indicators of socially aversive or schizotypal personality traits and adverse childhood experiences, whereas the EI measure should show larger unique effects for indicators of adaptive psychological adjustment.

## Results

### Correlations among the study measures

We observed a high correlation (*r* = .77, *p* < .001) between the general factors of the OPD-SQ and the TEIQUE. This correlation was homogenous and not influenced by particular data points or scale levels (see Appendix Figure S1). At a subfactor level (see Appendix Table S1), the correlations within the two inventories were similar to those between the two inventories, with the median intercorrelation among the OPD-SQ scales being *r* = .56, median intercorrelation of the TEIQUE subscales *r* = .49, and median intercorrelation between the two inventories being *r* = .46. Both measures were highly correlated with self-esteem (r = .69 and *r* = .71, ps < .001, respectively), given both conceptualizations encompass aspects of self-esteem. Controlling the correlation between OPD-SQ and TEIQUE for self-esteem yielded a partial correlation of *r* = .56 (p < .001). Note that this analysis is performed at a latent level in the following section. Concerning validity measures included in this study, OPD-SQ structural integration and TEIQUE EI displayed highly similar correlation profiles. We further investigated the common and specific portions of variance that the two constructs share with these validity measures in commonality analyses (see below).

### Latent variable models

We set up structural equation models to investigate (a) the latent correlation among the two constructs and (b) selfesteem as a possible confounding variable. In a first step, we tested the fit of the single measurement models, before entering them in a joint structural model (Anderson & Gerbing, 1988). To this end, we were interested in the correlations between general rather than specific factors between the two constructs (as both constructs have different factor structures, but probably the same general factor).

The OPD-SQ model converged to an admissible solution and displayed good fit to the data (*χ* ^2^(2) = 3.86, *p* = .15; CFI = 0.995; RMSEA= 0.075 (*P*_RMSEA<.05_=.26); SRMR= 0.014). Standardized factor loadings ranged from *β* = .76 to β = .87 and were statistically significant (p < .001). Also, the TEIQUE model estimation converged to an admissible solution and the model showed good fit to the data (*χ* ^2^(2) = 1.34, *p* = .51; CFI = 1.000; RMSEA= 0.000 (*P*_RMSEA<.05_=.64); SRMR= 0.013). Factor loadings ranged from *β* = .56 to *β* = .85 (p < .001). For self-esteem, the fit of the initial measurement model was not as good as for the two main models (*χ* ^2^(9) = 29.15, *p* < .001; CFI = 0.958; RMSEA= 0.116 (*P*_RMSEA<_.05=.01); SRMR= 0.038). Modification indices suggested the specification of a residual correlation between self-esteem for physical attractiveness and sportiness, which is theoretically meaningful as the two body-related scales are seen as closely related aspects of selfesteem by the authors (Schütz et al., 2016). We thus included this residual correlation (*r* = .34), which substantially improved the model fit (*χ* ^2^(8) = 12.79, *p* = .12; CFI = 0.99; RMSEA= 0.06 (*P*_RMSEA<.05_=.34); SRMR= 0.025). Factor loadings ranged from *β* = .56 to *β* = .86 (p < .001).

To estimate the latent correlations between the OPD-SQ and the TEIQUE general factors, we set up a joint model for the two constructs. Model fit was not as good as for the single measurement models, but rather shows deterioration due to joint modeling of two highly related constructs as correlated but distinguishable^2^ (*χ* ^2^(19) = 77.62, *p* < .001; CFI = 0.922; RMSEA= 0.137 (*P*_RMSEA<.05_=.00); SRMR= 0.048). The latent correlation between the OPD-SQ and the TEIQUE was estimated at *r* = .92 (*p* < .001), thus indicating almost perfect convergence between the two constructs.

**Figure 1:**
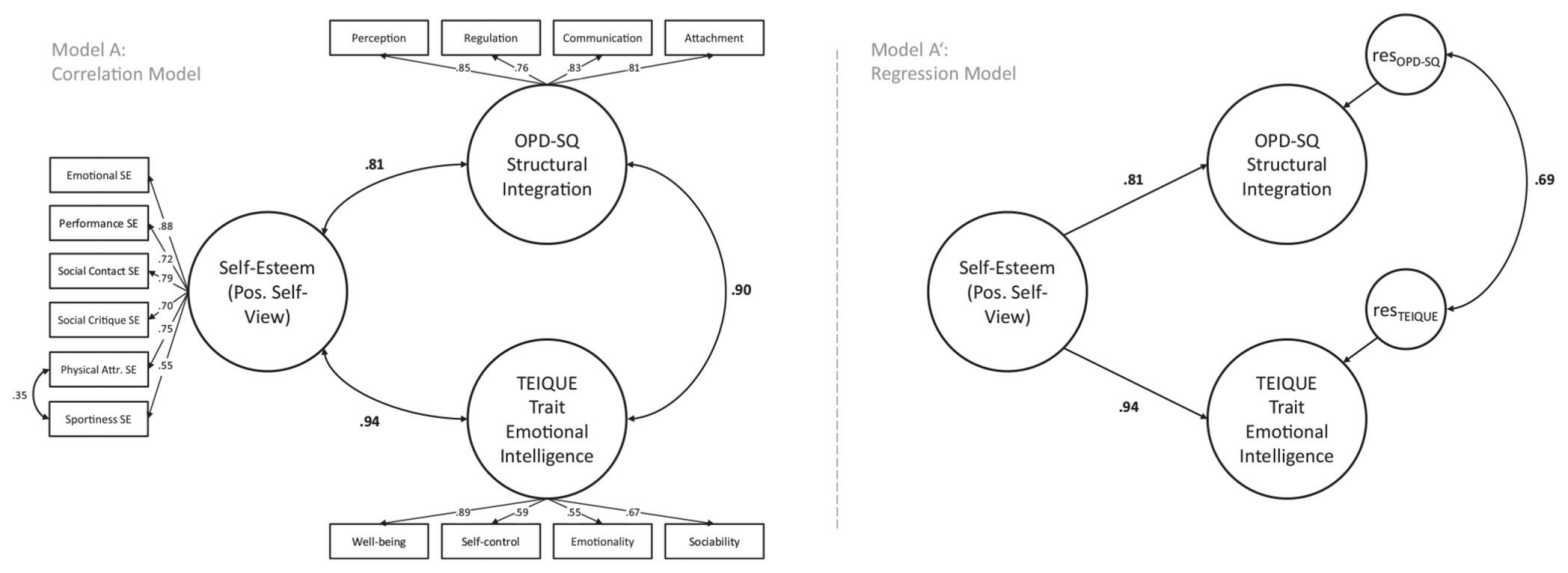
Latent variable structural equation models for investigating the relationship between general factors of structural integration and Trait Emotional Intelligence (Study 1) at zero order (model A: correlation model, left) and controlling for self-esteem (model A’: regression model, right). For ease of Interpretation, OPD-SQ scores were reversed so that higher scores indicate higher structural integration. OPD-SQ = Operationalized Psychodynamic Diagnosis – Structure Questionnaire, TEIQUE = Trait Emotional Intelligence Questionnaire, SE = self-esteem. Error terms are not displayed. Loadings of indicator variables are not displayed in model A’ as they equal those of model A.

To test for the potentially confounding effect of the general positivity of self-views, operationalized as self-esteem, we tested two equivalent models of the relations among the three constructs. In model A, self-esteem was correlated with both constructs. In Model A’, self-esteem was specified as a predictor of both constructs. The difference in the correlations between the two factors in model A and their residuals (controlling for self-esteem) in model A’ allows to test the extent by which the general positivity of self-views can account for the correlation between OPD-SQ and TEIQUE general factors. The model converged to an admissible solution. Again, model fit was not as good as for the separate models, and deterioration is likely due to modeling highly correlated constructs as correlated but distinguishable (*χ* ^2^(73) = 235.09, *p* < .001; CFI = 0.893; RMSEA= 0.116 (*P*_RMSEA<.05_=.00); SRMR= 0.058). The latent correlation between OPD-SQ and TEIQUE general factors was estimated at *r* = .90 (*p* < .001) in model A. The correlations between self-esteem and the two variables were also very high (*r_self-esteem_*, OPD-SQ = ^.81^, *r_self-esteem_*, TEIQUE = .94; see Figure 1). In model A’, where self-esteem was held constant, the latent correlation between OPD-SQ and TEIQUE factors was estimated at *r* = .69 (p < .001). Thus, while controlling for self-esteem markedly reduced the latent correlation, it was still substantial.

### Commonality analyses

To estimate the unique and shared amounts of variance in relation to the criterion measures included in this study, we performed commonality analyses. We used variables as criteria that also displayed significant zero-order correlations with the two constructs (which was not the case for narcissism and Machiavellianism). Results (see Table 2) show that the shared variance is the strongest factor for most criteria, but beyond that, the OPD-SQ has higher unique contributions to explaining clinically oriented personality measures/experienced adversity, whereas the TEIQUE has higher unique contributions to measures of adaptive functioning. For unusual experiences and adverse childhood experiences, the unique contributions of the OPD-SQ were even stronger than the shared effects. Study 3 will provide further commonality analyses encompassing broader nonclinical and clinical personality measures.

## Study 2

### Method

#### Participants and procedure

The sampling procedure and sample characteristics were similar to study 1. A total of *N* = 204 (101 male, 103 female) individuals took part in study 2. The sample size was chosen in order to detect small effects (*r* ~ .20) in zero-order correlations at a power of 1 – *β* = .8 and to allow for the estimation of structural equation models (*N* ~ 200 or at least 5 per estimated parameter; Kline, 2011). The mean age was 24.26years (*SD* = 3.56; range 20 – 40). All were native German speakers (96.6%) or had sufficient German skills (as assessed by the experimenters) to take part in the study. 0.5% had less than 12 years of schooling, 73.0% had at least 12 years of schooling or professional education, and 26.5% had a university degree. 29.9% of the sample were students of psychology, the rest had diverse study majors or professions. Participants gave written informed consent; the study protocol was approved by the IRB of the University of Graz (Austria). The sample reported here partially overlaps with a previous study, where other aspects were analyzed (Jauk et al., 2017; Study I).

As in study 1, participants were approached by students of the University of Graz (Austria) using standardized test booklets. Similar to study 1, participants completed measures of personality, childhood experiences, sexuality, and EI. The relevant measures are described below. The order of assessment was: self-esteem, STEM, OPD-SF, Dark Triad. The study duration was about 45 minutes. The percentage of missing data did not exceed 2.0% (*n* = 4) on any item. Data can be obtained via the Open Science Framework: https://osf.io/dcmeq/.

#### Measures

##### OPD structural integration

As in study 1, we assessed the level of structural integration using the OPD-SQ (Ehrenthal et al., 2012). Similar to study 1, the overall internal consistency of the general factor was *α* = .92.

##### Ability emotional intelligence

We assessed *emotion management* – the highest branch of EI according to performance models – using the Situational Test of Emotion Management (STEM; Austin, 2010; MacCann & Roberts, 2008). While the original STEM consists of 40 situational judgment items, we used an abbreviated German version consisting of 20 items. A sample item of the STEM is: *Lee’s workmate fails to deliver an important piece of information on time, causing Lee to fall behind schedule also. What action would be the most effective for Lee?* The test-taker is then presented four response alternatives (in this case: *work harder to compensate*/*get angry with the workmate*/*explain the urgency of the situation to the workmate/never rely on that workmate again)*, of which one has to be chosen (in this case the third alternative is considered correct).

Itemwise binary scoring (correct/incorrect) was performed based on the expert ratings provided by MacCann and Roberts (2008). The mean of the abbreviated STEM (see Appendix Table S2) shows that participants solved on average 63% of the items correctly, indicating adequate average item difficulty for the sample. The internal consistency was *α* = .57 at manifest level, indicating rather low reliability of the abbreviated scale. However, we aimed for a latent model of Ability EI, which turned out to be satisfactory (see below).

#### Personality

##### Self-esteem

As in study 1, we assessed self-esteem using the German Multidimensional Self-Esteem Scale (Schütz et al., 2016). Internal consistency was *α* = .82.

##### Dark triad traits

Also as in study 1, we assessed narcissism, Machiavellianism, and psychopathy using the short Dark Triad Dirty Dozen measure (Jonason & Webster, 2010). Internal consistencies of the short scales were *α* = .73, *α* = .77, and *α* = .67, similar to study 1.

#### Analysis strategy

We first analyzed correlations between the study variables at manifest level. Then, as in study 1, we set up structural equation models for the correlation between OPD structural integration and Ability EI. Again, we first evaluated separate measurement models for OPD structural integration and Ability EI, and then tested the structural relation between both constructs. Modeling of OPD structural integration conformed exactly to study 1. For modeling the STEM, we used the 20 items as indicators and weighted least squares with mean and variance adjustment (WLSMV) estimation for dichotomous indicators.

## Results

### Correlations among the study measures

Means and correlations of the OPD-SQ and measures of self-esteem and personality were generally similar to study 1 (see Appendix Table S2). Concerning our research question on the relationship between structural integration and Ability EI, we observed a low (*r* = .15) but significant (*p* = .03) correlation at the global factor level. Relationships were stronger for the interpersonal factors *regulation of relationships* (*r* = .30, *p* < .001) and *object perception* (*r* = .21, *p* < .01).

### Latent variable models

The measurement model of OPD-SQ structural integration converged to an admissible solution and displayed good fit to the data. (*χ* ^2^(2) = 2.46, *p* = .29; CFI = 0.999; RMSEA= 0.034 (p_RM_sE_A_<.05=.45); SRMR= 0.011). Standardized factor loadings ranged from *β* = .68 to *β* = .94 and were statistically significant (p < .001). The measurement model of STEM emotion management (using WLSMV estimation for dichotomous indicators) also converged to an admissible solution and showed good fit to the data (*χ* ^2^(170) = 170.43, *p* = .48; CFI = 0.998; RMSEA= 0.004 (p_RMS_E_A_<.05=1.00); WRMR= 0.829). Though only 16 out 20 items displayed significant loadings (*β* = .25 to *β* = .72, *p* < .05) on the latent variable and the model could have been improved by exclusion of the remaining four items, we did not make any changes to the theoretically assumed model.

The joint model of OPD structural integration and ability emotional intelligence (using WLSMV estimation) also displayed good fit to the data (*χ* ^2^(251) = 242.16, *p* = .64; CFI = 1.000; RMSEA= 0.000 (*P*RMSEA<.05=1.00); WRMR= 0.811). Appendix Figure S2 provides the estimates of the factor loadings (which are similar to the single measurement models) and the latent correlation of *r* = .20 (p = .02), which indicates a significant relationship between OPD structural integration and Ability EI.

## Study 3

### Method

#### Participants and procedure

The aim of study 3 was to replicate and extend the previous results in a community sample. We conducted an online survey on Amazon’s Mechanical Turk (www.mturk.com) administered via limesurvey (www.limesurvey.org). The targeted sample size was *N*> 300 to detect small effects at a power of 1 – *β* = .8 and to allow for the estimation of complex structural equation models (*N* ~ 200 or at least 5 per estimated parameter; Kline, 2011). The final sample consisted of *N*= 349 individuals (156 women, 192 men, 1 other; see Appendix S3 for exclusion criteria from initial sample of *N* = 402). The mean age was 36.28 years (*SD* = 11.55; range 18 – 73). All were US-residents and native English speakers. The highest attained education was high school for 18.6%, bachelors for 53.0%, masters for 25.5%, and doctoral degree for 0.9% of the sample. Participants reported diverse professions or study majors. 75.9% self-identified as caucasian, 11.2% as afro-american, 7.4% as hispanic/latino, 3.2% as asian, 0.9% as native american, and 1.4% as other. Participants gave written informed consent; an IRB approval is not required for fully anonymous self-report - research at our institutions. The study duration was on average 33 minutes (*SD* = 19). Participants received a monetary compensation of 3$. There was no missing data as all items were mandatory. Data can be obtained via the Open Science Framework: https://osf.io/dcmeq/.

### Measures

#### OPD structural integration

As in studies 1 & 2, we assessed the level of structural integration using the OPD-SQ (Ehrenthal et al., 2012). The overall internal consistency was *α* = .99; the eight subfactors ranged between *α* = .67 - .97.

#### DSM-5 personality functioning

We complemented the OPD-based assessment of structural integration with the 80-item self-report measure of personality functioning based on the DSM-5 AMPD (DSM-5 AMPD LPFS-SR: Morey, 2017). Internal consistencies of the four subfactors *identity*, *self-direction*, *empathy*, and *intimacy* ranged between *α* = .88 and *α* = .92, the overall internal consistency was *α* = .98.

#### Trait emotional intelligence

As in Study 1, we assessed trait EI using the TEIQUE (Petrides, 2009). The overall internal consistency was *α* = .97; the subfactors ranged from *α* = .82 to *α* = .93.

#### Ability emotional intelligence

As in Study 2, we assessed emotion management – the highest branch of EI – using the abbreviated STEM (MacCann & Roberts, 2008). Items and scoring correspond exactly to Study 2. The mean (see Table S3) indicates that, on average, participants solved 45% of the items correctly. The internal consistency of the measure was satisfactory with *α* = .76.

### Personality

#### Self-esteem

We assessed self-esteem using the 10-item Rosenberg self-esteem scale (RSES; Rosenberg, 1965). The internal consistency of the scale was *α* = .85.

#### Five-factor model nonclinical

We used the 30-item short form of the Big Five Inventory – 2 (Soto & John, 2017) to assess nonclinical Five-Factor Model personality dimensions. The internal consistencies for the 6-item scales were: neuroticism (negative emotionality) *α* = .77, extraversion *α* =.60, openness (open-mindedness) *α* = .73, agreeableness *α* = .72, and conscientiousness *α* = .75.

#### Five-factor model clinical

We used the 25-item brief form of the Personality Inventory for DSM-5 (American Psychiatric Association, 2013; see also Anderson et al., 2018) to assess clinical Five-Factor Model personality dimensions. The internal consistencies for the 5-item scales were: negative affect (neuroticism) *α* = .87, detachment (introversion) *α* =.89, psychoticism (~ openness) *α* = .91, antagonism (disagreeableness) *α* = .90, and disinhibition (low conscientiousness) *α* = .90.

### Socially desirable responding

We assessed socially desirable response style using a 10-item forced-choice form of the Marlowe-Crowne Social Desirability Scale (MCSDS; Crowne & Marlowe, 1960; form X1 according to Fischer & Fick, 1993). We coded the nondesirable option 0 and the socially desirable option 1. The mean was 0.40 (see Table S3), indicating that, on average, participants agreed with four of the statements. Internal consistency of the scale was rather low with *α* = .44.

### Analysis strategy

**Figure 2:**
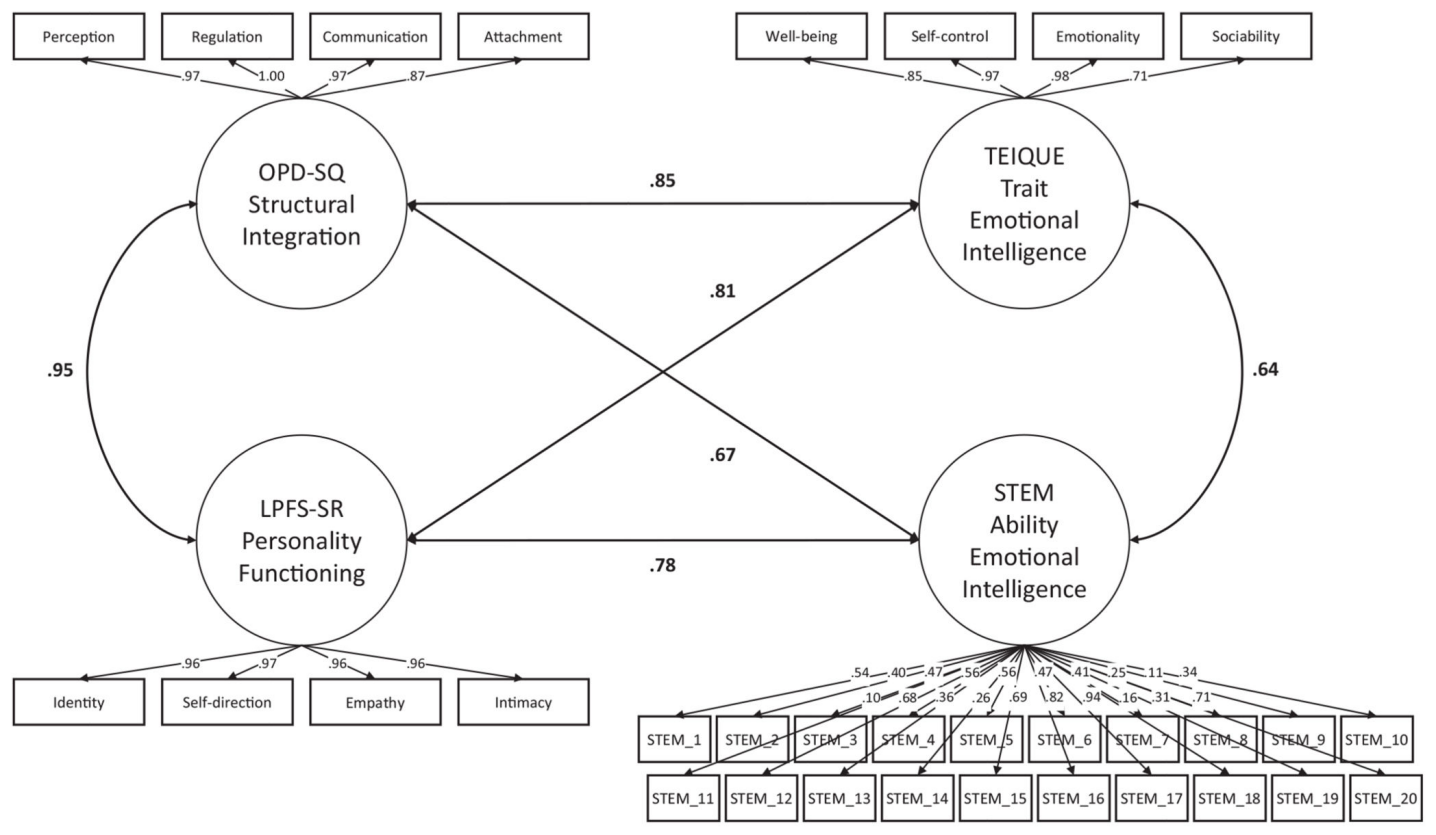
Latent associations of structural integration and personality functioning with Trait Emotional Intelligence and Ability Emotional Intelligence (Study 3). For ease of Interpretation, OPD-SQ and LPFS-SR scores were reversed so that higher scores indicate higher structural Integration / personality functioning. OPD-SQ = Operationalized Psychodynamic Diagnosis – Structure Questionnaire, LPFS-SR = Level of Personality Functioning Scale – Self-Report, TEIQUE = Trait Emotional Intelligence Questionnaire, STEM = Situational Test of Emotion Management. Error terms are not displayed.

As in studies 1 and 2, we first evaluated manifest correlations among OPD structural integration – complemented by DSM-5 AMPD personality functioning – and Trait as well as Ability EI. Again, we controlled these for potential confounds, namely self-esteem and also socially desirable responding. Next, we analyzed the latent relationships among measures of structural integration/personality functioning and Trait as well as Ability EI. Modeling corresponded exactly to studies 1 and 2; the DSM-5 AMPD LPFS-SR was modeled along the four subfactors identity, self-direction, empathy, and intimacy (see Figure 2), conforming to the AMPD structure. Finally, as in study 1, we used commonality analyses to investigate the shared and unique variance with nonclinical and clinical personality scales.

## Results

### Correlations among the study measures

The means and *SD*s (see Appendix Table S3) generally indicate that the community sample reported higher levels of structural impairment/lower levels of EI alongside higher variance in these measures than the previous two samples. Similarly, the STEM mean was lower, and variance was higher. The correlation of *r* = .82 (*p* < .001) between the OPD-SQ and the TEIQUE as an indicator of Trait EI was similar to study 1. Again, the general factor correlation was stronger than correlations at subfactor level (median intercorrelation *r* = .69). Regarding Ability EI, we observed a substantially higher correlation of *r* = .58 (p < .001) here than in the other two samples, which is likely due to higher variance (see discussion). Also, we observed a near-perfect correlation between the OPD-SQ and the LPFS-SR (*r* = .94, *p* < .001), supporting their concurrent validity, and very high correlations between the LPFS-SR and the TEIQUE (*r* = .78, *p* < .001) as well as the STEM (r = .68, *p* < .001).

The correlation patterns of structural integration/personality functioning and Trait EI factors and subfactors with self-esteem (RSES) were of generally comparable magnitude to those observed in study 1 (see Table S3). Partial correlations controlling for self-esteem yielded a similar picture as in study 1 (*r_OPD-SF, TEIQUE; RSES_* = .56, *p* < .001; *r_OPD-SF, STEM; RSES_* = .47, *p* < .001). Correlations with socially desirable responding (MCSDS) ranged from *r* = .10 to *r* = .44 and were generally higher for the two measures of structural integration/personality functioning than for the TEIQUE.

Controlling for socially desirable responding did not markedly alter the correlations (*r_OPD-SF, TEIQUW; MCSDS_* = .81, *p* < .001; *r_OPD-SF, STEM; MCSDS_* = .55, *p* < .001).

### Latent variable models

The measurement models of the three self-report scales converged to admissible solutions and displayed largely acceptable data fit^3^ (OPD-SQ: *χ* ^2^(2) = 14.73, *p* < .001; CFI = 0.995; RMSEA= 0.135 (*P*_RMSEA<.05_=.01); SRMR= 0.005, standardized loadings from *β* = .90 to *β* = .98; LPFS-SR: *χ* ^2^(2) = 12.36, *p* < .01; CFI = 0.995; RMSEA= 0.122 (*P*_RMSEA<_.05=.03); SRMR= 0.004, standardized loadings from *β* = .96 to *β* = .97; TEIQUE: *χ* ^2^(2) = 2.50, *p* = .29; CFI = 1.000; RMSEA= 0.027 (*P*_RMSEA<.05_=.55); SRMR= 0.006, standardized loadings from *β* = .80 to *β* = .92). The measurement model of the STEM (using WLSMV estimation) also showed acceptable data fit (*χ* ^2^(170) = 256.57, *p* = .00; CFI = 0.946; RMSEA= 0.038 (*P*_RMSEA<.05_=0.98); WRMR= 0.990; standardized loadings from *β* = .10 to *β* = .94, with two insignificant loadings). The joint model (using WLSMV estimation) converged and showed acceptable data fit (*χ* ^2^(458) = 605.26, *p* = .00; CFI = 0.991; RMSEA= 0.030 (*P*_RMSEA<.05_= 1.00); WRMR= 0.771).

Figure 2 displays the joint model. The Latent correlation of *r* = .85 (p < .001) between the OPD-SQ and the TEIQUE was similar to study 1, the latent correlation with the STEM was again markedly higher (reflecting the manifest correlation pattern; *r* = .67, *p* < .001). We observed similar results for the DSM-5 AMPD LPFS-SR, which shows that the results generalize to personality functioning as conceptualized in the DSM-5.

### Commonality analyses

As in study 1, we investigated the relative contributions to explained variance in scales designed to measure primarily adaptive or maladaptive aspects of personality. We used nonclinical and clinical Five-Factor Model scales. As Table 2 shows, commonality analyses showed that – while shared variance is almost always highest – the relative contribution of the OPD-SF is higher when explaining clinical personality variation, whereas the relative contribution of the TEIQUE is higher when explaining nonclinical personality variance.

## Discussion

We tested the hypothesis that personality functioning, assessed by the OPD-SQ, a self-report measure of the LSIA of the OPD system, and EI – particularly Trait EI – could be closely related constructs at a general level. We derived this hypothesis from the considerable theoretical and empirical overlaps in the literatures on both constructs.

### Structural integration and trait emotional intelligence

In line with our hypotheses, in study 1, we observed a high correlation (*r* = .77) between self-report structural integration and Trait EI at a manifest level, and an almost perfect correlation at a latent level (*r* = .90). These results replicated in a community sample in study 3 (manifest *r* = .82, latent *r* = .85) Importantly, the correlation was neither readily attributable to the general positivity of self-views – operationalized via self-esteem – which can be a major source of covariance among personality items containing evaluative content (Leising et al., 2015), nor to socially desirable responding. Study 3 further showed that the association generalizes to a different measures of personality functioning (DSM-5 AMPD LPFS-SR manifest *r* = .78, latent *r* = .81), which further indicates convergence across different conceptualizations of structural integration/personality functioning.

The correlation between structural integration and Trait EI was strongest at a general factor level, whereas the correlations at the level of single factors – though substantial – were markedly lower. This is in line with our expectation to observe a very high degree of correspondence only at the most general level, when disregarding the specific structures of the LSIA and Trait EI models. While these structures are overlapping in some aspects (for instance the LSIA factor *self-regulation* with the TEIQUE factor *self-control*, which correlated at *r* = .61 in study 1 and *r* = .85 in study 3), in others, the models make different assumptions about (a) where in the factor space specific abilities or traits should be placed, (b) whether and how they should be parted into more fine-grained aspects (which is more the case in the OPD as in the Trait EI system), and (c) which of them should go together. For instance, while intra- and interpersonal affect/emotion perception is partially assigned to the factors *self-perception* (affect differentiation), *object perception* (self-object differentiation), and *internal communication* (experiencing affect) in the OPD system, it is represented in the emotion perception (self and others) facet of the *emotionality* subfactor in the TEIQUE model. These differences might have emerged from a process view, on the one hand, and an individual differences view on the other. Our study is neither an attempt to test these models against each other, nor to synthesize them, though the latter could be an interesting future field of study. For now, we believe it is important to demonstrate that both models assess similar latent constructs, which might have implications for the study of human emotional competencies, as discussed in more detail below.

Regarding criterion validity with respect to individual differences constructs, we hypothesized that OPD-SQ structural integration and TEIQUE Trait EI scores would have mostly shared effects, but display specificity depending on whether validity indicators assess primarily maladaptive or adaptive qualities. Specifically, we expected that the OPD-SQ would show stronger covariance with clinical personality variation and adverse experiences, whereas the TEIQUE would show stronger unique effects on adaptive adjustment measures and nonclinical personality variation. Commonality analyses largely confirmed this hypothesis. While the shared effects were strongest in most cases, OPD-SQ structural integration showed stronger unique contributions to the prediction of psychopathy, unusual (psychotic-like) experiences, adverse childhood experiences, and all Five-Factor Model dimensions when assessed with a clinically oriented scale (personality inventory for DSM-5). The TEIQUE, in contrast, displayed stronger unique contributions to the prediction of perceived social support, life satisfaction, and all Five-Factor Model dimensions when assessed with a nonclinical personality inventory. These analyses thus indicate that both, structural integration and Trait EI can be placed on the same continuum of personality functioning, but have different predictive qualities at the lower and upper bounds of this continuum.

### Structural integration and ability emotional intelligence

In studies 2 and 3, we investigated the relations of OPD-SQ structural integration and Ability EI, as measured by the STEM (MacCann & Roberts, 2008). The STEM assesses the highest branch of Ability EI, as proposed in Mayer and Salovey’s (1997) hierarchical model, using a situational judgment paradigm. In study 2, we observed a low to moderate, significant latent correlation of *r* = .20 between the two measures, similar in magnitude to those previously reported between measures of Trait and Ability EI (Brannick et al., 2009). In the community sample investigated in study 3, the correlation was markedly higher (manifest *r* = .58, latent *r* = .67). We observed a similar correlation for the DSM-5 LFPS (manifest *r* = .68, latent *r* = .78). The higher correlations observed in study 3 could result from higher variance in structural integration/personality functioning scales and STEM performance (with a notable number of subjects performing below chance level, which might indicate deliberate choice of maladaptive emotion management strategies). However, the results await replication in future studies, and the “true” estimate might lie in between those obtained in studies 2 and 3. For now, it can be concluded that there is substantial correlation, which shows that relations between structural integration/personality functioning and EI extend from self-perceptions (of “typical performance”) to Ability EI measures (indicative of “maximal performance”) to a sizeable degree.

### Implications for research on structural integration and emotional intelligence

Though our findings are preliminary in nature and await replication and extension (see below) in future studies, we believe the high degree of correspondence between selfreport measures of structural integration/personality functioning (OPD-SQ and DSM-5 AMPD LPFS-SR) and Trait EI (TEIQUE) has important implications for both research traditions. The near-perfect latent correlations between the two constructs indicate that these assess highly overlapping latent dimensions, and the two areas of research – though originating in different academic and applied traditions – might have more in common than one might expect at first glance. If this would hold true in future studies, it would mean that (a) empirical findings from the two research traditions could generalize from one to the other, allowing for a transfer of knowledge from the social/personality psychology field to the clinical field, and vice versa. Both have unique strengths, such as the rigorous quantitative methodology and large-scale studies on the one hand, and the precise, nuanced, and in-depth observation and theory building on the other. Ultimately, an exchange between the two research traditions might stimulate (b) the development of integrative models of human emotional competencies.

The findings might also add to the growing body of literature on personality functioning as conceptualized in the DSM-5 AMPD (Bender et al., 2011) or the ICD-11 (Tyrer et al., 2019). While the OPD LSIA can be conceived an indicator of personality functioning (Zimmermann et al., 2012), which is also reflected in the joint analysis in study 3, it has, to our knowledge, not been proposed that EI might also be considered an indicator of personality functioning (though impaired EI has been associated with borderline personality disorder in some studies; e.g. Peter et al., 2013). A further promising step might be to integrate research on the GFP – which was also found to be highly overlapping, if not identical with Trait EI (van der Linden et al., 2017) – with contemporary models of personality functioning. The frequently encountered critique that general factor models reflect artifacts of response behavior rather than true common variance (cf. Revelle & Wilt, 2013) could be evaluated by adding performance-based measures of Ability EI and clinician-assessed levels of personality functioning.

Taken together, our findings add to evidence that general personality functioning is a viable individual differences concept that is evident across different models from clinical and personality psychology. Cross-validation and integration of these models may contribute to a comprehensive and integrative understanding of human personality.

### Limitations and future directions

Our findings could be extended in several ways: First, while our studies provide first evidence for the investigated hypothesis, future studies could use clinical samples, study longitudinal associations, or the influence of contextual factors to gain a closer understanding of whether, when, and under which conditions convergence between measures of structural integration/personality functioning and EI can be observed. Second, we targeted correspondence between structural integration and EI at a general level, but did not perform facet- or item-level analyses of the OPD-SQ and the TEIQUE. These might reveal similarities and differences in factor structures, which might provide suggestions for joint factor solutions which could be validated using measures of Ability EI or clinician ratings of structural integration. Third, we focused on self-report measures of structural integration/EI (studies 1 and 3), which not only share method variance, but also reflect “experience-near” assessments of the respective constructs. In contrast, research on the OPD LSIA uses expert-ratings, which are seen as gold standard in the OPD system. Even when taking into account a substantial correlation of *r* = .62 between OPD-SQ and LSIA expert-ratings (Dinger et al., 2014), we cannot rule out that the high degree of correspondence is to some extent due to shared self-report method variance. Though we controlled for the positivity of self-views and socially desirable responding, only a cross-validation with expert-ratings will reveal whether the constructs overlap irrespective of the assessment method. Finally, we note that the associations of the STEM with self-report scales were unexpectedly high in study 3. This might have different reasons, from higher variance (and more low-scorers) in this sample, to the online administration of the STEM in which no experimenter is present to double-check that participants correctly understand the instructions. Future studies could use more ecologically valid measures of emotionally intelligent behavior, for instance ecological momentary assessment in real-life situations. This could also unveil situational factors which might foster or hinder the enactment of EI in everyday life.

## Conclusion

We tested the hypothesis that structural integration, as assessed by the OPD-SQ, and Trait EI, as assessed by the TEIQUE, might be highly overlapping or isomorphic constructs at a general level. High manifest correlations (*r* = .77 - .82) and near-perfect latent correlations (*r* = .85 - .90) between both constructs confirmed this hypothesis. Correlations with validity measures were mostly due to the shared variance among both, but displayed specificity regarding clinical/nonclinical constructs. Structural integration was further substantially related to Ability EI (*r* = .20 - .65). The findings provide evidence for the concept of general personality functioning across clinical and nonclinical models.

## Supplementary Material

Supplemental material

## Figures and Tables

**Table 1:** Models of OPD structural integration and TEIQUE trait emotional intelligence.

OPD Structural Integration		
	*Self*	*Object*	TEIQUE Emotional Intelligence
Perception	Self-Perception	Object-Perception	Well-Being
	self-perception affect differentiation sense of identity	self-object-differentiation holistic object perception realistic object perception	self-esteem trait happiness trait optimism
Regulation	Self-Regulation	Regulation of Relationships	Self-Control
	affect tolerance impulse control regulation of self-esteem	protecting relationships^+^ balancing interests^+^ anticipation	emotion control stress management impulse control
Emotional Communication	Internal	External	Emotionality
	experiencing affect use of fantasies bodily self	establishing contact communicating affect empathy	emotion perception (self and others) emotion expression relationships trait empathy
Attachment	To Internal Objects	To External Objects	Sociability
	internalization use of introjects variability of attachment^-^	capability for attachment^-^ accepting help detaching from relationships	social awareness emotion management (others) assertiveness (Not loading on a factor) adaptability self-motivation

*Note*. Facets marked with ^–^ are not included in the OPD-SQ; facets marked with ^+^ are tied together in the single facet “protecting interests in relationships” in the OPD-SQ. Note that OPD and TEIQUE factor structure overlap only in some aspects but differ in others (see text for details). OPD-SQ = Operationalized Psychodynamic Diagnosis – Structure Questionnaire, TEIQUE = Trait Emotional Intelligence. Questionnaire

**Table 2:** Commonality analyses of unique and shared variance of structural integration and trait emotional intelligence on selected clinical personality scales and adaptive/maladaptive psychological functioning (study 1) as well as clinical and nonclinical five-factor model personality dimensions (study 3).

	OPD-SQ Structural Integration Unique	Shared Variance	TEIQUE Trait Emotional Intelligence Unique
*Study 1*			
**Personality Clinical**			
Psychopathy	.16	.77	.07
Unusual Experiences	.69	.23	.08
**Adaptive Functioning**			
Perceived Social Support	.01	.69	.30
Life Satisfaction	.08	.77	.15
**Maladaptive Functioning**			
Adverse Childhood Experiences	.68	.24	.08
*Study 3*			
**Personality Clinical**			
Negative Affect	.23	.76	.01
Detachment	.22	.77	.02
Psychoticism	.46	.52	.02
Antagonism	.36	.64	.00
Disinhibition	.32	.68	.00
**Personality Nonclinical**			
Neuroticism (Negative Emotionality)	.01	.75	.24
Extraversion	.16	.11	.73
Openness (Open-Mindedness)	.04	.79	.17
Agreeableness	.07	.81	.12
Conscientiousness	.06	.81	.14

Note. Coefficients in each line add up to 1. OPD-SQ = Operationalized Psychodynamic Diagnosis – Structure Questionnaire, TEIQUE = Trait Emotional Intelligence Questionnaire.

## Footnotes

1 While the main focus of this work will be on *Trait EI*, we nonetheless move from Ability to Trait EI in introducing these constructs, which is more in line with the conceptual progression of the respective research traditions.

2 Deviations from the data are likely not due to misspecification of either of the models, but rather due to the joint modeling of two highly related constructs without specification of either a general factor or indicator-level correlations. We nonetheless had a-priori interest in this model to obtain an estimate of the latent correlation which is unbiased by indicator-level correlations.

3 Note that while some coefficients, above all *χ* ^2^, indicate worse fit than in studies 1 and 2, the larger sample size in study 3 also implies higher power for deviation tests. Parameter estimates are highly similar to studies 1 and 2.
