# Supplementary material for: Self-Reported Levels of Personality Functioning from the Operationalized Psychodynamic Diagnosis (OPD) System and Emotional Intelligence Likely Assess the Same Latent Construct
Source: J Pers Assess. Author manuscript; Available in PMC 2021 Jul 17. (PMC7611281; doi:10.1080/00223891.2020.1775089)
Supplement: Supplemental material [file EMS130371-supplement-Supplemental_material.zip › TableS1.docx]

Table S1. Descriptive statistics and correlations of study 1 measures.

|  | *M* (*SD*) | 2 | 3 | 4 | 5 | 6 | 7 | 8 | 9 | 10 | 11 | 12 | 13 | 14 | 15 | 16 | 17 | 18 | 19 | 20 | 21 | 22 |
| --- | --- | --- | --- | --- | --- | --- | --- | --- | --- | --- | --- | --- | --- | --- | --- | --- | --- | --- | --- | --- | --- | --- |
| OPD-SQ Structural Integration (1) | 1.19 (0.42) | .82 | .75 | .81 | .62 | .50 | .81 | .86 | .75 | .77 | .74 | .58 | .55 | .49 | .69 | -.01 | -.06 | -.25 | -.45 | .41 | .56 | -.25 |
| Self-perception (2) | 0.87 (0.59) |  | .57 | .71 | .41 | .47 | .58 | .67 | .50 | .67 | .65 | .51 | .45 | .46 | .57 | .03 | -.04 | -.16 | -.39 | .33 | .46 | -.13 |
| Object perception (3) | 1.18 (0.47) |  |  | .47 | .53 | .24 | .60 | .65 | .47 | .51 | .48 | .47 | .35 | .31 | .41 | -.13 | -.14 | -.23 | -.34 | .24 | .36 | -.19 |
| Self-regulation (4) | 1.14 (0.54) |  |  |  | .52 | .34 | .63 | .62 | .54 | .67 | .64 | .61 | .39 | .47 | .70 | .06 | .02 | -.11 | -.38 | .34 | .46 | -.20 |
| Regulation of relationships (5) | 1.31 (0.52) |  |  |  |  | .09 | .52 | .40 | .29 | .47 | .34 | .37 | .47 | .27 | .32 | -.13 | -.24 | -.35 | -.33 | .18 | .22 | -.16 |
| Internal communication (6) | 0.94 (0.46) |  |  |  |  |  | .24 | .42 | .26 | .49 | .44 | .23 | .46 | .32 | .41 | .11 | .09 | -.10 | -.04 | .31 | .29 | -.07 |
| External communication (7) | 1.41 (0.53) |  |  |  |  |  |  | .64 | .61 | .64 | .63 | .34 | .47 | .51 | .62 | .08 | .05 | -.20 | -.38 | .25 | .46 | -.19 |
| Attachment to internal objects (8) | 1.07 (0.66) |  |  |  |  |  |  |  | .63 | .63 | .67 | .48 | .42 | .31 | .56 | -.05 | -.09 | -.19 | -.40 | .43 | .59 | -.33 |
| Attachment to external objects (9) | 1.60 (0.74) |  |  |  |  |  |  |  |  | .53 | .54 | .41 | .33 | .28 | .50 | -.03 | -.02 | -.15 | -.38 | .35 | .47 | -.21 |
|  |  |  |  |  |  |  |  |  |  |  |  |  |  |  |  |  |  |  |  |  |  |  |
| TEIQUE Emotional Intelligence (10) | 5.05 (0.51) |  |  |  |  |  |  |  |  |  | .85 | .67 | .76 | .77 | .71 | .10 | .03 | -.24 | -.26 | .49 | .59 | -.15 |
| Well-being (11) | 5.51 (0.80) |  |  |  |  |  |  |  |  |  |  | .49 | .50 | .60 | .74 | .19 | .07 | -.13 | -.26 | .46 | .68 | -.25 |
| Self-control (12) | 4.64 (0.59) |  |  |  |  |  |  |  |  |  |  |  | .35 | .36 | .49 | -.06 | -.10 | -.07 | -.29 | .31 | .36 | -.14 |
| Emotionality (13) | 5.24 (0.65) |  |  |  |  |  |  |  |  |  |  |  |  | .45 | .30 | -.04 | -.17 | -.50 | -.17 | .49 | .35 | -.09 |
| Sociability (14) | 4.96 (0.67) |  |  |  |  |  |  |  |  |  |  |  |  |  | .62 | .28 | .32 | .03 | -.09 | .29 | .40 | -.03 |
|  |  |  |  |  |  |  |  |  |  |  |  |  |  |  |  |  |  |  |  |  |  |  |
| Self-Esteem (15) | 4.78 (0.94) |  |  |  |  |  |  |  |  |  |  |  |  |  |  | .14 | .18 | .03 | -.21 | .26 | .42 | -.15 |
|  |  |  |  |  |  |  |  |  |  |  |  |  |  |  |  |  |  |  |  |  |  |  |
| Dark Triad |  |  |  |  |  |  |  |  |  |  |  |  |  |  |  |  |  |  |  |  |  |  |
| Narcissism (16) | 3.28 (1.33) |  |  |  |  |  |  |  |  |  |  |  |  |  |  |  | .47 | .26 | .21 | -.06 | .12 | .07 |
| Machiavellianism (17) | 3.02 (1.23) |  |  |  |  |  |  |  |  |  |  |  |  |  |  |  |  | .46 | .07 | -.11 | .02 | .07 |
| Psychopathy (18) | 2.28 (1.05) |  |  |  |  |  |  |  |  |  |  |  |  |  |  |  |  |  | .15 | -.19 | -.11 | .05 |
|  |  |  |  |  |  |  |  |  |  |  |  |  |  |  |  |  |  |  |  |  |  |  |
| Unusual Experiences (19) | 0.24 (0.21) |  |  |  |  |  |  |  |  |  |  |  |  |  |  |  |  |  |  |  |  |  |
|  |  |  |  |  |  |  |  |  |  |  |  |  |  |  |  |  |  |  |  | -.17 | -.32 | .24 |
| Psychological Adjustment |  |  |  |  |  |  |  |  |  |  |  |  |  |  |  |  |  |  |  |  |  |  |
| Perceived Social Support (20) | 6.10 (0.86) |  |  |  |  |  |  |  |  |  |  |  |  |  |  |  |  |  |  |  | .44 | -.30 |
| Life Satisfaction (21) | 5.21 (1.06) |  |  |  |  |  |  |  |  |  |  |  |  |  |  |  |  |  |  |  |  | -.38 |
|  |  |  |  |  |  |  |  |  |  |  |  |  |  |  |  |  |  |  |  |  |  |  |
| Adverse Childhood Experiences (22) | 3.57 (2.14) |  |  |  |  |  |  |  |  |  |  |  |  |  |  |  |  |  |  |  |  |  |

Note. *N* = 166. Correlations exceeding *r* = .15, .20, and .25 are significant at *p* < .05, .01, and .001, respectively. OPD-SQ = Operationalized Psychodynamic Diagnosis - Structure Questionnaire. TEIQUE = Trait Emotional Intelligence Questionnaire. Correlations between the OPD-SQ and the other measures are inversed (higher score indicates higher levels of structural integration) for ease of interpretation. Means of the OPD-SQ correspond to the original scoring (higher score indicates higher structural impairment).
