# Supplementary material for: Self-Reported Levels of Personality Functioning from the Operationalized Psychodynamic Diagnosis (OPD) System and Emotional Intelligence Likely Assess the Same Latent Construct
Source: J Pers Assess. Author manuscript; Available in PMC 2021 Jul 17. (PMC7611281; doi:10.1080/00223891.2020.1775089)
Supplement: Supplemental material [file EMS130371-supplement-Supplemental_material.zip › TableS2.docx]

Table S2. Descriptive statistics and correlations of study 2 measures.

|  | *M* (*SD*) | 2 | 3 | 4 | 5 | 6 | 7 | 8 | 9 | 10 | 11 | 12 | 13 | 14 |
| --- | --- | --- | --- | --- | --- | --- | --- | --- | --- | --- | --- | --- | --- | --- |
| OPD-SQ Structural Integration (1) | 1.34 (0.42) | .83 | .76 | .81 | .72 | .57 | .65 | .79 | .72 | .15 | .49 | -.16 | -.13 | -.16 |
| Self-perception (2) | 1.15 (0.68) |  | .50 | .75 | .46 | .51 | .51 | .54 | .51 | .05 | .51 | -.08 | -.06 | .02 |
| Object perception (3) | 1.35 (0.47) |  |  | .52 | .64 | .29 | .44 | .65 | .48 | .21 | .20 | -.26 | -.18 | -.29 |
| Self-regulation (4) | 1.19 (0.56) |  |  |  | .52 | .37 | .42 | .54 | .55 | .08 | .53 | -.11 | -.05 | .02 |
| Regulation of relationships (5) | 1.45 (0.56) |  |  |  |  | .36 | .35 | .52 | .39 | .30 | .16 | -.29 | -.39 | -.42 |
| Internal communication (6) | 1.06 (0.45) |  |  |  |  |  | .30 | .41 | .23 | .07 | .36 | -.00 | -.02 | -.18 |
| External communication (7) | 1.54 (0.51) |  |  |  |  |  |  | .41 | .43 | -.01 | .46 | .01 | -.03 | .02 |
| Attachment to internal objects (8) | 1.32 (0.65) |  |  |  |  |  |  |  | .52 | .15 | .35 | -.12 | -.13 | -.24 |
| Attachment to external objects (9) | 1.64 (0.67) |  |  |  |  |  |  |  |  | .08 | .28 | -.10 | .05 | .05 |
|  |  |  |  |  |  |  |  |  |  |  |  |  |  |  |
| STEM Ability Emotional Intelligence (10) | 12.65 (2.96) |  |  |  |  |  |  |  |  |  | -.11 | -.08 | -.26 | -.26 |
|  |  |  |  |  |  |  |  |  |  |  |  |  |  |  |
| Self-Esteem (11) | 4.87 (0.79) |  |  |  |  |  |  |  |  |  |  | .10 | .09 | .21 |
|  |  |  |  |  |  |  |  |  |  |  |  |  |  |  |
| Dark Triad |  |  |  |  |  |  |  |  |  |  |  |  |  |  |
| Narcissism (12) | 3.66 (1.17) |  |  |  |  |  |  |  |  |  |  |  | .32 | .22 |
| Machiavellianism (13) | 2.90 (1.22) |  |  |  |  |  |  |  |  |  |  |  |  | .54 |
| Psychopathy (14) | 2.68 (1.15) |  |  |  |  |  |  |  |  |  |  |  |  |  |

Note. *N* = 204. Correlations exceeding *r* = .14, .18, and .23 are significant at *p* < .05, .01, and .001, respectively. OPD-SQ = Operationalized Psychodynamic Diagnosis - Structure Questionnaire. STEM = Situational Test of Emotion Management. Correlations between the OPD-SQ and the other measures are inversed (higher score indicates higher levels of structural integration) for ease of interpretation. Means of the OPD-SQ correspond to the original scoring (higher score indicates higher structural impairment).
