# Supplementary material for: Self-Reported Levels of Personality Functioning from the Operationalized Psychodynamic Diagnosis (OPD) System and Emotional Intelligence Likely Assess the Same Latent Construct
Source: J Pers Assess. Author manuscript; Available in PMC 2021 Jul 17. (PMC7611281; doi:10.1080/00223891.2020.1775089)
Supplement: Supplemental material [file EMS130371-supplement-Supplemental_material.zip › TableS3.docx]

Table S3. Descriptive statistics and correlations of study 3 measures.

|  | *M* (*SD*) | 2 | 3 | 4 | 5 | 6 | 7 | 8 | 9 | 10 | 11 | 12 | 13 | 14 | 15 | 16 | 17 | 18 | 19 | 20 | 21 | 22 |
| --- | --- | --- | --- | --- | --- | --- | --- | --- | --- | --- | --- | --- | --- | --- | --- | --- | --- | --- | --- | --- | --- | --- |
| OPD-SQ Structural Integration (1) | 1.85 (0.93) | .96 | .97 | .96 | .97 | .86 | .94 | .95 | .77 | .94 | .93 | .91 | .88 | .91 | .82 | .75 | .81 | .77 | .61 | .58 | .74 | .43 |
| Self-perception (2) | 1.73 (1.24) |  | .93 | .93 | .93 | .86 | .87 | .88 | .65 | .92 | .90 | .91 | .88 | .88 | .78 | .69 | .77 | .77 | .59 | .62 | .70 | .40 |
| Object perception (3) | 1.89 (1.10) |  |  | .91 | .95 | .80 | .91 | .91 | .70 | .93 | .91 | .90 | .88 | .90 | .74 | .66 | .75 | .71 | .54 | .59 | .67 | .44 |
| Self-regulation (4) | 1.73 (1.00) |  |  |  | .92 | .85 | .87 | .89 | .71 | .91 | .91 | .90 | .86 | .87 | .83 | .76 | .85 | .77 | .63 | .58 | .76 | .40 |
| Regulation of relationships (5) | 1.85 (1.13) |  |  |  |  | .81 | .90 | .90 | .70 | .91 | .90 | .89 | .87 | .88 | .77 | .69 | .78 | .74 | .55 | .60 | .68 | .43 |
| Internal communication (6) | 1.67 (0.74) |  |  |  |  |  | .74 | .77 | .52 | .83 | .81 | .84 | .79 | .79 | .75 | .66 | .71 | .76 | .59 | .61 | .66 | .33 |
| External communication (7) | 1.96 (0.93) |  |  |  |  |  |  | .89 | .77 | .85 | .85 | .82 | .80 | .83 | .75 | .69 | .74 | .68 | .59 | .46 | .68 | .41 |
| Attachment to internal objects (8) | 1.95 (1.15) |  |  |  |  |  |  |  | .75 | .89 | .88 | .85 | .83 | .87 | .78 | .77 | .74 | .73 | .55 | .48 | .75 | .40 |
| Attachment to external objects (9) | 1.99 (0.74) |  |  |  |  |  |  |  |  | .65 | .66 | .61 | .58 | .66 | .65 | .63 | .65 | .56 | .48 | .30 | .60 | .34 |
|  |  |  |  |  |  |  |  |  |  |  |  |  |  |  |  |  |  |  |  |  |  |  |
| LPFS-SR Overall (10) | 319.92 (115.65) |  |  |  |  |  |  |  |  |  | .98 | .98 | .96 | .97 | .78 | .72 | .78 | .76 | .55 | .68 | .68 | .39 |
| Identity (11) | 98.54 (34.34) |  |  |  |  |  |  |  |  |  |  | .94 | .91 | .92 | .76 | .70 | .78 | .71 | .53 | .63 | .67 | .39 |
| Self-Direction (12) | 77.11 (31.07) |  |  |  |  |  |  |  |  |  |  |  | .94 | .92 | .78 | .70 | .77 | .76 | .57 | .68 | .66 | .38 |
| Empathy (13) | 56.49 (21.52) |  |  |  |  |  |  |  |  |  |  |  |  | .92 | .75 | .67 | .74 | .74 | .54 | .68 | .62 | .37 |
| Intimacy (14) | 87.77 (32.02) |  |  |  |  |  |  |  |  |  |  |  |  |  | .75 | .71 | .73 | .74 | .52 | .66 | .67 | .38 |
|  |  |  |  |  |  |  |  |  |  |  |  |  |  |  |  |  |  |  |  |  |  |  |
| TEIQUE Emotional Intelligence (15) | 4.47 (0.82) |  |  |  |  |  |  |  |  |  |  |  |  |  |  | .92 | .92 | .93 | .84 | .56 | .79 | .23 |
| Well-being (16) | 4.55 (1.07) |  |  |  |  |  |  |  |  |  |  |  |  |  |  |  | .79 | .82 | .70 | .45 | .82 | .19 |
| Self-control (17) | 4.44 (0.96) |  |  |  |  |  |  |  |  |  |  |  |  |  |  |  |  | .80 | .71 | .57 | .70 | .28 |
| Emotionality (18) | 4.59 (0.92) |  |  |  |  |  |  |  |  |  |  |  |  |  |  |  |  |  | .74 | .61 | .71 | .22 |
| Sociability (19) | 4.34 (0.74) |  |  |  |  |  |  |  |  |  |  |  |  |  |  |  |  |  |  | .36 | .63 | .10 |
|  |  |  |  |  |  |  |  |  |  |  |  |  |  |  |  |  |  |  |  |  |  |  |
| STEM Ability Emotional Intelligence (20) | 9.02 (4.13) |  |  |  |  |  |  |  |  |  |  |  |  |  |  |  |  |  |  |  | .39 | .24 |
|  |  |  |  |  |  |  |  |  |  |  |  |  |  |  |  |  |  |  |  |  |  |  |
| Self-esteem (21) | 2.88 (0.64) |  |  |  |  |  |  |  |  |  |  |  |  |  |  |  |  |  |  |  |  | .21 |
|  |  |  |  |  |  |  |  |  |  |  |  |  |  |  |  |  |  |  |  |  |  |  |
| Socially desirable responding (22) | 0.40 (0.19) |  |  |  |  |  |  |  |  |  |  |  |  |  |  |  |  |  |  |  |  |  |

Note. *N* = 349. Correlations exceeding *r* = .10, .14, and .17 are significant at *p* < .05, .01, and .001, respectively. OPD-SQ = Operationalized Psychodynamic Diagnosis - Structure Questionnaire. LPFS-SR = Levels of Personality Functioning Scale - Self Report. STEM = Situational Test of Emotion Management. Correlations between the OPD-SQ as well as the LPFS-SR and the other measures are inversed (higher score indicates higher levels of structural integration / personality functioning) for ease of interpretation. Means of the OPD-SQ and the LPFS-SR correspond to the original scoring (higher score indicates higher structural impairment).

Of the initial sample of *N* = 402, we excluded participants who did not pass at least one of two attention checks (*n* = 20), took less than 10 minutes to complete the survey (additional *n* = 12), or indicated that their data should not be analyzed because they did not provide accurate answers (additional *n* = 4). We further excluded individuals whose mother tongue was not English (additional *n* = 4), who provided free-text answers indicative of not answering the survey properly (additional *n* = 11), or showed no variation in responses^[[1]](#footnote-1)^ to the TEIQUE (additional *n* = 2).

1. Within this sample, we also observed a strong peak in the distribution of the TEIQUE scores, which was not evident in the other inventories (I.e., an unexpectedly high numbers of participants displayed average TEIQUE scores, but varied on the OPD-SQ and the LPFS-SR). These participants, however, did not show any noticeable response patterns (such as no variation between items). To evaluate whether they influenced the correlational results, we also conducted the analyses without these participants, but obtained highly similar results. Thus, they were retained in the sample. [↑](#footnote-ref-1)
